# Screening for STIs: Results of a Health-Promotion Programme in a Portuguese University

**DOI:** 10.3390/microorganisms12122479

**Published:** 2024-12-02

**Authors:** Joana M. Oliveira, Ana Helena Martins, Daniela Veiga, Célia Lavaredas, António Queirós, Ana Miguel Matos

**Affiliations:** 1 CERES, Faculty of Pharmacy, University of Coimbra, 3000-548 Coimbra, Portugal; joana.oliveira@student.ff.uc.pt; 2 Laboratory of Microbiology, Faculty of Pharmacy, University of Coimbra, 3000-548 Coimbra, Portugal; helena.ana82@gmail.com; 3 Centre for Functional Ecology (CFE), Associate Laboratory TERRA, Department of Life Sciences, University of Coimbra, 3000-548 Coimbra, Portugal; 4 Laboratory of Clinical Analysis from University of Coimbra—LACUC, 3000-548 Coimbra, Portugal; daniela.ff.veiga@gmail.com; 5 Serviços de Saúde e de Gestão da Segurança no Trabalho, University of Coimbra, 3000-548 Coimbra, Portugal; celia.lavaredas@sas.us.pt (C.L.); aqueiros@sas.uc.pt (A.Q.)

**Keywords:** STI, university, chlamydia, gonorrhoea, syphilis, *Mycoplasma genitalium*

## Abstract

Sexually Transmitted Infections (STIs) are an important and growing public health concern. Implementation of screening programmes and awareness campaigns are crucial to mitigate this problem. A university in the central region of Portugal has devised a health-promotion programme, named *Protection+*, specifically directed towards the sexual health of the university community. The present study aimed to evaluate the results of the different actions undertaken as part of the health-promotion programme during the 2023–2024 academic year. Chlamydia, gonorrhoea, trichomoniasis and infection with *Mycoplasma genitalium* were assessed through real-time polymerase chain reaction protocols. Syphilis, infection with HIV, HBV and HCV were assessed through immunological assays. The adherence to the health-promotion programme after the awareness campaigns was also evaluated. STIs have been diagnosed in 13.7% of the 475 screened participants. Chlamydia was the STI most frequently diagnosed (8.4%), followed by infection with *M. genitalium* (2.3%), *T. pallidum* (2.0%) and *N. gonorrhoeae* (1.1%). HIV, HBV and HCV were diagnosed in a residual number of cases, and *T. vaginalis* was not detected in any of the screened participants. At the time of diagnosis, more than half of the infected patients were asymptomatic. After the implementation of awareness campaigns, an increase in the adherence to STI screening was observed, with the expected simultaneous increase in STI diagnoses. The high prevalence of STIs, particularly chlamydia, in the university population, along with the asymptomatic nature of these infections, demonstrated the importance of STI screenings and the implementation of campaigns that raise awareness on the prevention and consequences of untreated STIs.

## 1. Introduction

Sexually Transmitted Infections (STIs) represent a global health concern, with a significant impact on sexual and reproductive health worldwide [1,2].

STIs can be caused by a variety of microorganisms, including bacteria, viruses, protozoa and parasites [2,3]. According to the World Health Organisation (WHO), the majority of global STI cases are associated with eight specific pathogens [4]. Four of these infections are currently curable, namely chlamydia, gonorrhoea, syphilis and trichomoniasis [5]. The remaining four are associated with viral infections without effective treatment, and they include the herpes simplex virus, hepatitis B virus (HBV), human immunodeficiency virus (HIV) and human papillomavirus (HPV). Of these, two are preventable through vaccination (HBV and HPV), and the remaining can be controlled through available antiviral molecules [6,7,8,9].

Emerging STI agents have been identified. Infection with *Mycoplasma genitalium* has been increasingly reported in the general population [10,11]. Hepatitis A virus (HAV), monkeypox virus, Ebola virus and Zika virus outbreaks have been described in specific high-risk groups, such as men who have sex with men (MSM) [12,13,14,15].

In developed countries, the most prevalent STIs are chlamydia, gonorrhoea, syphilis and trichomoniasis [16]. Nevertheless, viral STIs should not be overlooked, since their acquisition may be increased in the presence of non-viral STIs [17].

Several factors increase the risk of STIs, namely earlier onset of sexual activity, the existence of multiple sexual partners and the incorrect use of condoms [18,19]. Various reports point to higher prevalence of STIs among young women [10,20], which can be explained by sexually risky behaviours in combination with the composition of vaginal mucosa, which is more susceptible to microbial invasion [16].

Recent European Centre for Disease Prevention and Control (ECDC) reports suggest that a considerable number of STIs remain undiagnosed in numerous countries, including Portugal. The primary reason for such an underdiagnosis is the asymptomatic nature of most STIs. Additionally, the lack of screening programmes and the frequent stigmatisation associated with STIs also contribute to the underdiagnosis of these infections, potentializing their transmission, as well as the development of long-term serious conditions including cancer, chronic pelvic pain, ectopic pregnancies and infertility [16,18,21,22].

Despite the availability of efficacious therapeutic options for certain STIs, the absence of diagnosis precludes the possibility of initiating treatment. Therefore, early diagnosis and the subsequent treatment of these infections are essential for the control of the transmission and the prevention of the associated complications [3,17]. In order to control the spread of STIs, the WHO recommended, along with education and prevention campaigns, the implementation of screening programmes, particularly among young individuals who are at an increased risk of acquiring such infections [3,21].

Recognising the importance and impact of STIs among the younger population, namely those attending higher education institutions, the Clinical Analysis Laboratory and the Health Services of a Portuguese university have devised a health-promotion programme, named *Protection+* (*Proteção+* in Portuguese), specifically designed for the university community. This pilot programme was designed and implemented in the academic year of 2023–2024.

The first action of the *Protection+* programme was the invitation of university members to take a screening test for various STIs. Although STI screenings are carried out in some Portuguese universities, these actions are often designed to screen only for HIV, HBV and HCV infections, excluding the aforementioned most common causes of STIs in developed countries (chlamydia, gonorrhoea, syphilis and trichomoniasis). The WHO has reported an increase in these STIs in recent years, but no updated data exist regarding the Portuguese population. Furthermore, the knowledge of the nature and extent of STIs among the university community facilitates the development of awareness campaigns that are better directed towards this specific population. Therefore, the *Protection+* programme was designed to screen for *Chlamydia trachomatis*, *Neisseria gonorrhoeae*, *Treponema pallidum*, *Trichomonas vaginalis*, *M. genitalium*, HIV, HBV, HCV and HAV. The inclusion of HAV in the screening was not intended to diagnose acute infection, but rather to assess the immunisation status of individuals with risky behaviours for acquiring such an infection through sexual relations and suggest vaccination to vulnerable individuals. Additionally, other sexually transmitted microorganisms, namely *Ureaplasma parvum*, *Ureaplasma urealyticum*, and *Mycoplasma hominis*, were also evaluated. Despite being considered part of the genital flora, the association of these microorganisms with a number of serious health problems has been hypothesised [23,24,25].

Based on the screening results, the health-promotion programme assured the follow-up of all the participants by the university clinical team, who prescribed appropriate treatment whenever needed and evaluated its effectiveness.

The health-promotion programme further comprised an STI awareness campaign. This campaign occurred during May 2024, when an important and popular academic celebration takes place. This period was chosen due to the higher probability of risky behaviours associated with this type of academic festivities. Therefore, the purposes of this campaign were to raise awareness on prevention, symptoms and treatment of STIs, as well as to remind the academic members of the opportunity offered by the university to screen for these infections. The campaign integrated various forms of advertising, including posters in the city and neighbouring towns, as well as several contents on digital platforms.

One year after the implementation of the *Protection+* health-promotion programme, the main objective of present study was to evaluate the results of the different actions undertaken, answering the following questions:

- How common are STIs among university students?
- What are the most common causes of STIs in the university population?
- Are the implemented treatments effective?
- Are the university members interested in STI screening?
- Is there an increase in participation in STI screening as a result of the awareness campaign?

The present study contributes to the monitoring of an increasingly important public health problem, and the obtained results stimulate future actions to reduce and control STIs among university communities.

## 2. Material and Methods

### 2.1. Set-Up of Protection+ Health-Promotion Programme

In September 2023, a university in the central region of Portugal launched the *Protection+* health-promotion programme, directed towards the sexual health of its members. This programme, relying on a screening campaign for STIs, was developed and adapted to the target population by the Health Services and the Clinical Laboratory Analysis of the university. An infographic publicising the programme was sent by e-mail, in October 2023, inviting all the members to participate in the screening.

The programme included the test for *C. trachomatis*, *N. gonorrhoeae*, *T. pallidum*, *T. vaginalis*, *M. genitalium*, HIV, HBV, HCV and HAV, and it started with a medical appointment, followed by sample collection.

The *Protection+* health-promotion programme included the follow-up of all the participants. Whenever necessary and applicable, treatment was initiated in accordance with the established national protocols and clinical guidelines. Antibiotic therapy was prescribed for *C. trachomatis*, *N. gonorrhoeae*, *M. genitalium* and *T. pallidum* infection, and a test-of-cure was recommended four weeks after treatment. Whenever infection with HIV, HBV or HCV was diagnosed, patients were referred to the Infectious Diseases Department of the University Hospital.

In May 2024, at the time of an important academic festivity, an STI awareness-raising campaign was launched in a different format, including publicity billboards and digital content.

### 2.2. Protection+ Participants

During the academic year 2023–2024, a total of 475 participants applied for the *Protection+* health-promotion programme. The group of participants, aged between 18 and 64 years (median age: 24 years), included 176 men (median age: 24 years) and 299 women (median age: 23 years) (Table 1). Information on symptoms related with STIs was gathered at the moment of sample collection. The participants were grouped according to gender and age (15–19; 20–24; 25–34; 35–44; ≥45 years), defined in accordance with ECDC reports. Follow-up appointments and respective samples were not considered for the present study. All the participants gave their informed written consent to the analysis performed.

### 2.3. Samples Collection and Processing

Urogenital samples, used for the screening of *C. trachomatis*, *N. gonorrhoeae*, *M. genitalium* and *T. vaginalis*, were collected from all 475 participants, comprising 289 vaginal swabs, four urethral swabs and 182 urine samples. Serum samples, used for the screening of HIV (N = 342), HBV (N = 336), HCV (N = 343), HAV (N = 295) and *T. pallidum* (N = 345) were only available for a part of the individuals.

#### 2.3.1. Urogenital Swabs

Urogenital swabs were collected by the clinician using Multi-Collect Specimen Collection Kit (Abbot^®^; Ref: 9K12-01, Abbott Laboratórios Lda, Amadora, Portugal). Upon arrival at the laboratory, each sample was properly vortexed and stored at −20 °C until nucleic acid extraction.

#### 2.3.2. Urine Samples

The first voided urine or occasional urine after at least one hour without urinating was collected by participants into sterile containers and delivered at the laboratory at the same day. Two aliquots of 1 mL of urine were centrifuged at 18,000× *g* for 10 min. The pellet was resuspended in 200 µL of saline solution and stored at −20 °C until nucleic acid extraction.

#### 2.3.3. Serum Samples

Blood samples were collected via venipuncture into tubes containing separation gel. Following blood clotting, samples underwent centrifugation for 20 min at 1500× *g*, serum was separated and stored at −20 °C until analysis.

### 2.4. STI Screening

All participants were screened for the presence of *C. trachomatis*, *N. gonorrhoeae* and *M. genitalium* using urogenital samples through real-time polymerase chain reaction (PCR) protocols. *T. vaginalis* and commensal microorganisms, *U. urealyticum*, *M. hominis* and *U. parvum* were assessed in urogenital samples from a part of the individuals (225 and 114, respectively), also through real-time PCR protocols. Nucleic acids were extracted from the processed urogenital swabs and urine samples using the QIAamp Viral Mini Kit (QIAGEN^®^ 52906, Werfen Portugal, Carnaxide, Portugal), according to the manufacturer’s instructions.

Three distinct real-time PCR protocols were used for the screening of STIs according to the available equipment and the university and laboratory policies.

Alinity^TM^ STI AMP Kit (Abbott^®^ Ref: 09N17-090, Abbott Laboratórios Lda, Amadora, Portugal ) was used for the genome detection of *C. trachomatis*, *N. gonorrhoeae*, *T. vaginalis* and *M. genitalium* on Alinity m^®^ equipment, according to the manufacturer’s instructions. TaqPath^TM^ GeneProof^TM^ CT/NG/MG Multiplex PCR Kit (Thermo Fisher Scientific^®^ Ref: A58117, Alfagene, Carcavelos, Portugal) was used for the genome detection of *C. trachomatis*, *N. gonorrhoeae* and *M. genitalium*. Allplex^TM^ STI Essestial Assay Q (MH, UU) (Seegene^®^ Ref: SD10201Y, Werfen Portugal, Carnaxide, Portugal) was used for genome detection of *C. trachomatis*, *N. gonorrhoeae*, *T. vaginalis*, *M. genitalium*, *M. hominis*, *U. parvum* and *U. urealyticum.*

Each amplification batch included positive, negative, and internal controls.

Whenever serum samples were available and consent by the patient was obtained, screening for infection with HIV, HBV, HCV and *T. pallidum* was carried out according to national guidelines (Table 1) [26,27,28]. The immunisation status of HAV infection was evaluated through the presence of specific antibodies.

Detection of antibodies and antigens of HIV, HCV, HBV and HAV was performed with an Enzyme-Linked Fluorescence Assay (ELFA) method, on VIDAS equipment (BioMérieux^®^, Linda-a-Velha, Portugal), according to the manufacturer’s instructions. Detection of HIV-1 and HIV-2 specific antibodies and p24 HIV-1 protein was performed using a 4th generation ELISA-based test (VIDAS^®^ HIV DUO Quick (HIV6) Ref: 30447, BioMérieux^®^, Linda-a-Velha, Portugal ). Detection of HCV specific antibodies was performed using VIDAS^®^ Anti-HCV (Ref: 30308; BioMérieux^®^, Linda-a-Velha, Portugal); HBV HBs antigen was evaluated with VIDAS^®^ HBs Ag Ultra (Ref 30315 BioMérieux^®^ ¸ Linda-a-Velha, Portugal) and HAV specific antibodies with VIDAS^®^ Anti-HAV Total (Ref: 30312; BioMérieux^®^, Linda-a-Velha, Portugal). Whenever positive results were obtained for HIV, HBV and HCV, confirmatory tests were conducted, according to national guidelines.

Syphilis screening was performed through the search of reagins on serum samples using the RPR test (Mascia Brunelli^®^ UC80600, Baptista Marques, Lisboa, Portugal), according to the manufacturer’s instructions. Positive results were subsequently validated through FTA-Abs test.

### 2.5. Statistical Analysis

GraphPad Prism (v. 10.3.1) was used to analyse both qualitative (categorical variables) and quantitative (continuous variables) data, and to generate graphic representations. Descriptive statistical analysis was used to summarise the characteristics of age data sets. Comparison of categorical variables was performed using the chi-square test. Fisher’s exact test was applied whenever any cell on the contingency table contained fewer than five expected observations. Student’s *t*-test was used for comparison between population groups; *p*-values below 0.05 were considered statistically significant.

## 3. Results

### 3.1. Protection+ Health-Promotion Programme Outcomes

**Figure 1:**
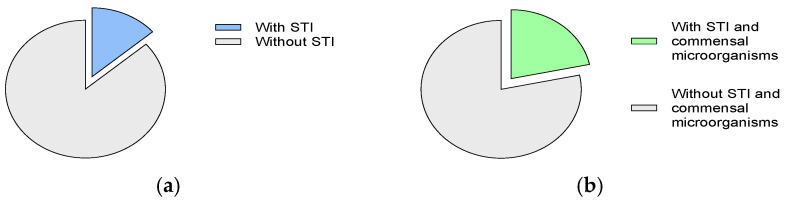
Frequency of detection of (**a**) STIs and (**b**) STIs and others sexually transmitted microorganisms.

During the academic year 2023–2024, a total of 475 participants, comprising 176 men and 299 women, attended the university STI screening campaign, which is part of the *Protection+* programme (Table 1). Sixty-five participants (13.7%) showed a positive result for at least one of the screened STI agents (*C. trachomatis, N. gonorrhoeae, M. genitalium, T. vaginalis,* HIV, HCV, HBV and *T. pallidum*) (Figure 1a). Chlamydia was the STI most frequently diagnosed, followed by infection with *M. genitalium* (2.3%), *T. pallidum* (2.0%) and *N. gonorrhoeae* (1.1%) (Table 1).

**Figure 2:**
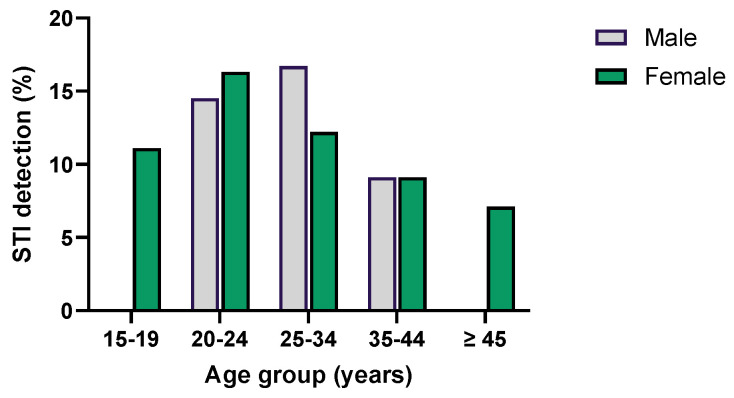
Distribution of STIs according to age group and gender.

STIs were diagnosed in 41 women (13.7%) and 24 men (13.6%) (*p* = 0.981). The median age of STI patients was 23 years (range: 19 to 53 years), with a higher frequency in individuals aged between 20 and 34 years (*p* = 0.539) (Figure 2).

In addition to those 65 participants with STI, another 38 (8.0%) were found to be infected with at least one commensal microorganism transmitted through sexual intercourse (Figure 1b). Twenty-four (36.9%) of the patients diagnosed with an STI and 16 (42.1%) of the participants infected only with commensal microorganisms presented some symptom suggestive of STI at the time of sample collection.

### 3.2. Infection with C. trachomatis

**Figure 3:**
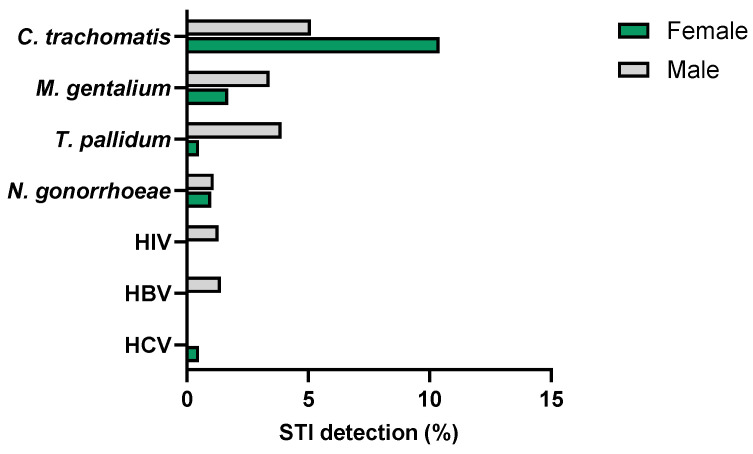
Prevalence of STIs according to gender.

Infection with *C. trachomatis* was detected in 40 of the 475 participants (8.4%), with a higher prevalence observed among those aged between 20 and 24 years (10.7%) (median age 22 years; range: 19 to 41 years) (Table 1). Chlamydia was more frequently diagnosed in women (31; 10.4%) than in men (9; 5.1%) (*p* = 0.046) (Figure 3), with a male-to-female ratio of 0.3. From the 40 participants diagnosed with chlamydia, only 16 (40.0%), including two men (22.2%) and 14 women (45.2%) (*p* = 0.272), were symptomatic at the time of sample collection.

### 3.3. Infection with M. genitalium

*M. genitalium* was identified in urogenital samples from eleven of the 475 (2.3%) participants (Table 1). The median age of the infected participants was 26 years (range: 20–53 years) (Table 1). Although infection with *M. genitalium* was diagnosed twice as often in men (3.4%) than in women (1.7%), the observed difference is not statistically significant (*p* = 0.224) (Table 1 and Figure 3). Only two of the infected participants (18.2%), both men, presented symptoms at the time of sample collection.

### 3.4. Infection with T. pallidum

Infection with *T. pallidum* was detected in 7 of 345 participants (2.0%). Syphilis patients included one woman (0.5%) and six men (3.9%) (*p* = 0.047) (Figure 3), aged between 20 and 31 years (median age: 24 years) (Table 1). Among the infected participants, only one (14.3%) man presented STI symptoms at the time of sample collection.

### 3.5. Infection with N. gonorrhoeae

*N. gonorrhoeae* was detected in 5 of the 475 (1.1%) participants, aged between 19 and 22 years (median age: 21). The infected participants included three women (1.0%) and two men (1.1%) (*p* = 0.891) (Table 1 and Figure 3). Only one (20%) man presented typical symptoms at the time of sample collection.

### 3.6. Infection with HIV, HBV and HCV

The diagnosis of HIV, HBV and HCV was only achieved in a residual number of participants. HIV infection was diagnosed in 2 of the 342 participants tested (0.6%), both men, aged 31 and 35 years. Moreover, 2 other men from the 336 participants, aged 28 and 29 years, were diagnosed with HBV infection (0.6%). HCV was only detected in 1 of the 343 participants (0.3%), a 35-year-old woman (Table 1 and Figure 3). None of these five individuals presented symptoms at the time of diagnosis.

### 3.7. Infection with U. parvum, U. urealyticum and M. hominis

**Figure 4:**
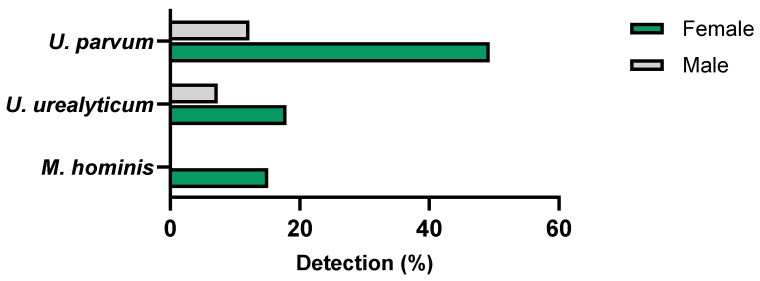
Prevalence of commensal microorganisms according to gender.

At least one of the evaluated commensal microorganisms was identified in 51 (44.7%) of the 114 screened participants. The median age of the participants with detectable commensal microorganisms was 24 years (range: 19 to 53 years). A significantly higher prevalence was observed among women than men (58.9% and 19.5%, respectively) (*p* < 0.05) (Figure 4).

Of these 51 participants, 13 (25.5%) were infected with one pathogenic agent. From the 38 individuals in which only commensal microorganisms were identified, 16 (42.1%) presented at least one symptom suggestive of STI at the time of sample collection, without significant differences between gender (*p* = 0.056).

The presence of *U. parvum* was observed in 41 participants (35.9%), including 36 women (49.3%) and 5 men (12.2%) (*p* < 0.05). *U. parvum* was detected in all age groups, with higher frequency in the age group of 25 to 34 years (median age: 24 years; range: 19 to 53 years). As for *U. urealyticum*, it was detected in 16 participants (14.0%), including 13 women (17.9%) and 3 men (7.3%) (*p* = 0.122), with a median age of 24 years (range: 19 to 51 years). *M. hominis* was identified in eleven participants, all female (15.1%), with a median age of 26 years (range: 20 to 35 years) (Table 1 and Figure 4).

### 3.8. Co-Detection of Multiple Agents

**Figure 5:**
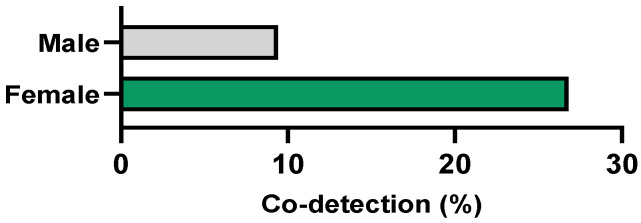
Prevalence of co-detection of multiple agents according to gender.

Of the 103 participants who tested positive for any of the screened sexually transmitted microorganisms (pathogenic and commensal), 22 (21.4%) presented the simultaneous detection of two or more agents (Table 2). These participants, 3 men (9.4%) and 19 women (26.8%) (*p* = 0.068), have a median age of 26.5 years (range: 19 to 41 years) (Figure 5).

The detection of two pathogenic agents occurred in three participants (Table 2), all men, aged between 25 and 34 years. None of these three coinfected participants were symptomatic at the point of sample collection.

Detection of one pathogenic agent and one or more commensal microorganisms was observed in 13 participants (Table 2), all female, with the median age of 25 years (range: 19 to 41 years). In 11 of these 13 cases, the pathogenic agent detected was *C. trachomatis*. In the remaining two cases, *M. genitalium* and HCV were the pathogenic agents present. Only 4 (30.8%) of these 13 participants presented STI symptoms at the time of the screening programme; nevertheless, no clear association could be deduced between symptoms and any of the microorganisms detected.

Detection of two or more commensal microorganisms was observed in six participants, with a median age of 25.5 years (range: 20 to 35 years) (Table 2). Four of these participants presented STI symptoms at the time of sample collection.

### 3.9. Immunity to HAV

**Figure 6:**
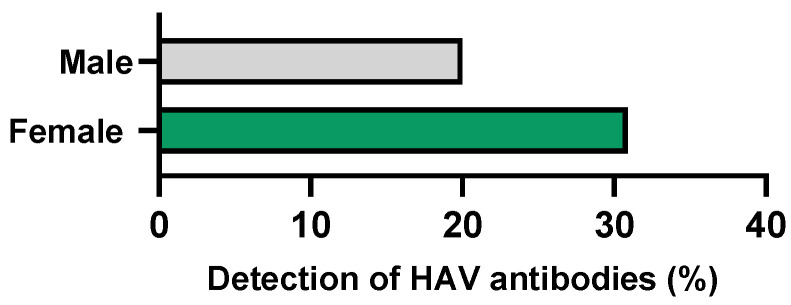
Frequency of detection of HAV-specific antibodies by gender.

HAV-specific antibodies were detected in 77 of the 295 (26.1%) participants tested. The median age of the participants immunised against HAV was 24 years (range: 18 to 45 years). A higher prevalence was observed in women (30.9%) than in men (20.0%) (*p* = 0.034) (Table 1 and Figure 6).

### 3.10. Follow-Up of STI Diagnosis

To assess the effectiveness of antibiotic treatment, a test-of-cure was recommended for the participants infected with *C. trachomatis*, *N. gonorrhoeae*, *M. genitalium* and *T. pallidum*. These microorganisms were detected in 60 participants, one of whom was coinfected with HIV and subsequently referred to the local hospital for treatment and follow-up. Of the remaining 59 participants, 16 (27.1%) proceeded with the test-of-cure which revealed that the implemented antibiotic treatment was effective in all patients.

### 3.11. Impact of Awareness Campaigns on the Participation in STI Screening

The target university community described in this study comprises nearly 34,000 members, of whom approximately 30,000 are students.

**Figure 7:**
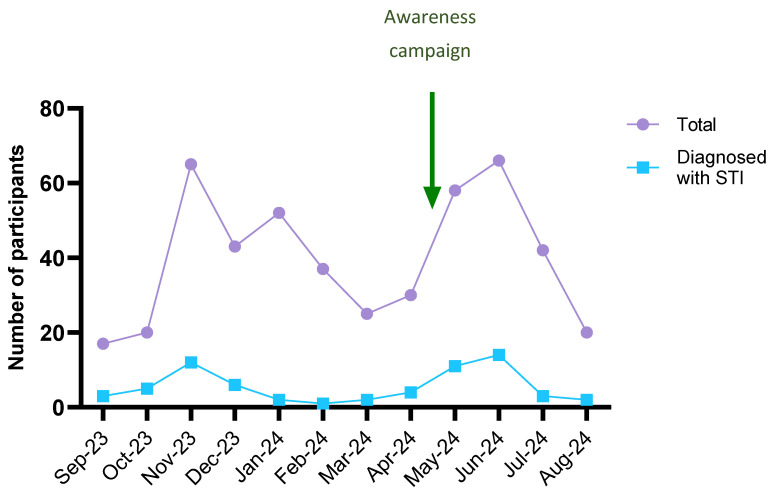
Evolution of the number of participants and STI cases during the academic year.

In September 2023, the STI screening programme was available to all the university members; however, the initial engagement was significantly lower than expected. Thus, in October 2023, an e-mail publicising the programme was sent to all university members. After this, the number of participants in the STI screening markedly increased, as well as the number of STI diagnoses (Figure 7). At the end of the first semester, the prevalence of STIs in the screened population was 12.4%. Nevertheless, a gradual decline in the number of participants was observed from January 2024 onwards. An awareness campaign was carried out in May 2024 during an important academic festivity. Similarly to October, there was a significant increase in participation in the screening programme and in the number of STI diagnoses (Figure 7).

## 4. Discussion

STIs are a significant public health problem due to their global and increasing prevalence, as well as the associated serious health complications [29]. The control of STIs represents one of the major concerns of the WHO, with screening and awareness programmes representing a crucial strategy for the achievement of this objective [3]. Nevertheless, some countries, including Portugal, have not implemented screening programmes for the most common STIs, namely *C. trachomatis*, *N. gonorrhoeae* and *T. vaginalis*.

Thus, the present study was designed to perform the first assessment of the prevalence of multiple STIs and other sexually transmitted commensal microorganisms in a Portuguese university population through the implementation of an innovative health-promotion programme, based on a complete STI screening. Despite the existence of numerous studies on the subject, the present study represents a pioneering investigation conducted on a specific Portuguese population group, known to be at an elevated risk of STIs [10,20]. The simultaneous screening of eight pathogenic agents and three commensal microorganisms further strengthen this study. Moreover, given that the majority of female samples selected for this study were vaginal swabs, a higher prevalence values was expected due to the significantly higher bacterial load in these cell-rich samples compared to urine samples [30,31]. Therefore, the results of this study contribute in clarifying the circulation of these agents among the university population and in overcoming the limited information on the real prevalence of infection with *C. trachomatis* and *M. genitalium*, for instance, in Portugal.

Nearly 14% of the studied population was diagnosed with an STI during the screening programme, without differences between gender. If commensal microorganisms sexually transmitted are also considered, the prevalence reaches a value of 22%. The obtained results are within the range of those reported in other studies. Several studies on the prevalence of pathogenic and commensal urogenital microorganisms have been published worldwide, but different prevalence values have been reported (1–38%, depending on the screened agent) [17,30,32,33,34,35]. These conflicting results are associated with multiple factors, namely the study population, age, gender and risky behaviours of the included individuals, as well as the methodology employed and the type of samples used [29]. As expected, in the present study, more than half of participants infected with at least one of the screened microorganisms were asymptomatic, which reinforces the importance of this type of screening programmes in this specific population group.

In this study, chlamydia was the STI most frequently diagnosed (8.4%), followed by infection with *M. genitalium* (2.3%), *T. pallidum* (2.0%) and *N. gonorrhoeae* (1.1%). The same profile of infection, but with slightly higher prevalence results (10.2%, 4.1% and 1.6%, respectively) were reported in the Netherlands by Nijhuis and colleagues (2021). Also, a study conducted among students (18 to 24 years) attending higher learning institutions in Tanzania reported a prevalence of 11% for *C. trachomatis* and 1.1% for *N. gonorrhoeae* [2,36]. The participants diagnosed with chlamydia were mostly aged between 20 and 24 years, which is in line with the age distribution described for European countries on the ECDC 2022 report, as well as with the results of other studies [2,17,37,38]. Also, chlamydia was more frequently diagnosed in women than in men. In fact, the male-to-female ratio obtained in the present study (0.3) shows an opposite distribution trend to that described in the ECDC 2022 report for Portugal (male-to-female ratio: 2.6) but is consistent with that described in the same report for European countries where STI screening and diagnosing programmes are already implemented (male-to-female ratio: 0.8) [37,39,40]. This discrepancy may be due to the targeted population and the lack of systematic screening of *C. trachomatis* infection. The impact of chlamydia among women has been well documented by several authors and prevalence values similar to that obtained in the present study have been reported across different countries [2,17,20,41]. Despite that symptomatology may be a reason for seeking medical care and participating in the screening programme, more than half of chlamydia patients were asymptomatic. This highlights the importance of screening, especially in a high-risk population group, such as university students.

In contrast to chlamydia, in Portugal, as in several other countries, *M. genitalium* infection is not a notifiable disease [42]. Consequently, official epidemiological data on this infection are scarce. In recent years, several studies have increasingly recognised *M. genitalium* as a cause of STI [11,43], leading to the inclusion of this infection in the present screening programme.

In fact, this microorganism was the second most frequent cause of STI (2.3%) in the present study. Higher prevalence values, ranging from 3.5% to 12.5%, have been reported in various countries, depending on the individuals and samples evaluated [11,40,44,45,46,47]. In Portugal, only a few studies on the prevalence of *M. genitalium* infection are available, with values ranging from 0.8% among Portuguese women of reproductive age [48], and 10.3% in Portuguese MSM [47]. Despite the median age of the infected participants being 26 years, different studies described infection with *M. genitalium* in all age groups, and the most affected group is not consistent across them [11,40,46]. The higher incidence of infection in men compared to women reported in this study was also observed by Spiller and colleagues (2020) [49]. Nevertheless, the majority of other studies reported that infection with *M. genitalium* was more common in women [45]. The results of the present study, together with previous studies, indicate an increase in the prevalence of infection with *M. genitalium* in the Portuguese population, providing further support for the inclusion of this bacterium in screening programmes.

Syphilis was the third most common STI diagnosed (2.0%). According to the ECDC 2022 report, the incidence of syphilis has increased in European countries in recent years, reaching a rate of 8.5 per 100,000 population in 2022. In Portugal, this increase is even more pronounced, with a rate of infection rising from 2.5 in 2018 to 14.8 in 2022 per 100 000 population [50]. An ECDC report further identifies a higher incidence of syphilis among men and in individuals aged between 25 and 34 years [50], which is consistent with the results of this study.

Similarly to syphilis, the incidence of gonorrhoea in Europe has been increasing in recent years [51]. As mentioned in the ECDC 2022 report, this trend is also observed in Portugal, with a rate of 21.8 per 100,000 population in 2022, slightly higher than the European mean rate (17.9 per 100,000 population) [51]. In the present study, gonorrhoea was diagnosed in 1.1% of the screened participants, which is in line with the prevalence values reported in other studies [2,36]. The young age of the infected participants (median age of 21 years) is in accordance with the distribution of this STI across the different age groups reported by the ECDC and other authors [51,52]. Despite the ECDC reporting a higher prevalence of gonorrhoea among men (male-to-female ratio: 4.2), with Portugal presenting an even higher male-to-female ratio [51], in the present study, no significant difference was observed between gender. Considering that gonorrhoea is frequently asymptomatic in women while men are more likely to present urogenital symptoms, it is anticipated that in the absence of screening programmes, as in Portugal, the number of reported cases will be higher for men once they seek medical advice [16].

STI screening programmes conducted among university communities frequently target HIV, HBV and HCV, as infections with these microorganisms are likely to be those that most concern the members of the university community. These programmes are normally based on point-of-care tests (POCT) and the results are only evaluated from an individual point of view. Nevertheless, the knowledge of the nature and extent of STI among the university community facilitate the development of awareness campaigns that are more directed towards this specific population. Therefore, the screening of these STI agents was also included in the current health-promotion programme but using ELISA-based methods instead of POCT, as those methodologies provide higher values of sensitivity and specificity.

In the present study, only a small number of participants were diagnosed with each of these STIs, namely two with HIV, two with HBV and one with HCV infections. According to the most recent ECDC 2023 report on HIV infection, incidence rates in Portugal have decreased in recent years (12.7 in 2018 to 7.8 in 2022 per 100,000 population); however, they remain higher than the mean European rate (5.1 per 100,000 population in 2022). The same ECDC report indicates that in Europe and, in particular, in Portugal, HIV most commonly affects men and individuals within the age group of 30 to 39 years, which is consistent with our results [53].

Recent studies have estimated a global prevalence of HBV infection between 3.2% to 3.8% [54,55]. Razavi-Schearer and colleagues (2023) further estimated a 1.1% prevalence of HBV infection in Portugal [54], which is a closer value to that observed in the present study (0.6%). HBV vaccine was introduced in the Portuguese National Vaccination Programme in 1995. Although a significant proportion of the Portuguese population, especially those over 30 years of age, is not immunised against this virus, the majority of the studied individuals were born after 1995, so it would be expected that they would be vaccinated, which may explain the low prevalence observed. Furthermore, despite all efforts to implement effective vaccination programmes, HBV infection remains highly prevalent in some geographic areas, including Africa and Western Pacific regions [56,57,58], with which Portugal has historical ties. The associated immigration process promotes the constant entry of non-vaccinated individuals in the country, which may represent a risk for the sexual transmission of this infection.

Contrarily to HBV, the prevalence value obtained for HCV infection (0.3%) is similar to that estimated for the global prevalence (0.8%) [55]. Considering the two liver pathogens evaluated in this study, HBV was detected more frequently than HCV (0.6% vs. 0.3%), a trend previously estimated by other authors [55]. The low prevalence observed for these STIs, is consistent with the data on ECDC 2022 reports, where the rate of infections in Portugal (1.5 per 100,000 population for HBV and 1.6 per 100,000 population for HCV) is lower than the European mean (8.5 per 100,000 population for HBV and 6.2 per 100 000 population for HCV) [57,59].

The presented findings, combined with the decline in infection rates reported by the ECDC for HIV, HBV and HCV, may reflect the control measures implemented [60].

According to the WHO, trichomoniasis is one of the four most common curable STIs worldwide [4]. However, its actual prevalence may be underestimated due to the fact that it is not a notifiable disease [16,42]. In light of these data, *T. vaginalis* infection was included in the present STI screening; nevertheless, trichomoniasis was not diagnosed in any of the participants. In fact, another study comprising 680 Portuguese women of reproductive age (15–44 years) reported a prevalence of only 1% for this pathogen [61], corroborating that this infection is, at present, uncommon among the Portuguese population, and it does not seem to represent a concern among the Portuguese group included in the present study. However, its importance should not be overlooked, and continuous monitoring of its circulation should be carried out. Associated complications of untreated *T. vaginalis* infections, mainly in pregnant women, further strength the need for such monitoring [16,62].

*U. parvum*, *U. urealyticum* and *M. hominis*, commonly known as commensal microorganisms, have been increasingly recognised as opportunistic pathogens of the urogenital tract in recent years [21,29]. The presence of these sexually transmitted microorganisms was also evaluated in the present screening programme, with almost 45% of the screened participants having one of these microorganisms in the urogenital tract. *U. parvum* was the most frequently detected (35.9%), followed by *U. urealyticum* (14.0%) and *M. hominis* (9.6%). Reported prevalence rates of these agents vary considerably between different countries [30,32,33,63]. Such discrepancies could be related with the target population, biological samples or methodologies applied for the microorganism detection [29]. Despite the discrepant values of prevalence, the microorganism distribution profile is similar across studies, with *Ureaplasma* species being more frequently detected than *M. hominis* [30,63,64,65,66]. Also, the majority of participants in whom these microorganisms were detected were aged between 20 and 34 years, which is consistent with the findings of previous studies [3,18,31,32].

In the present study, *M. hominis* was identified exclusively in samples from women, and *U. parvum* was found to be significantly more prevalent in women than in men, which is in accordance with the available literature [21,31]. Regarding *U. urealyticum*, although it was more frequent in women, no significant difference was observed between gender. The increased prevalence of these microorganisms in women may be partly related to the female anatomy, since female urogenital mucosa is thinner and more easily penetrated by infectious agents than the male urogenital mucosa [16]. At the time of sample collection, 42.1% of the participants with these agents present at least one STI-suggestive symptom. Considering these results along with the data available in the literature [21,31], these microorganisms should not be neglected, especially in the presence of symptoms. In fact, the presented data further support the hypothesis that these microorganisms should be considered as opportunistic pathogens rather than commensal microorganisms, especially when STI symptoms are present and no pathogenic microorganism is detected [29,30].

The detection of multiple agents (pathogenic and commensal) is a common feature reported in the literature. Damage in the urogenital mucosa associated with STIs provides an opportunity for the entry of other microorganisms [18,21,29,67,68]. Nearly one-fifth of participants with sexually transmitted agents were revealed to be infected with more than one microorganism. Despite not being statistically significant, the detection of two or more agents was more frequent in women than in men, probably due to the composition of the vaginal mucosa, which increases their vulnerability to microbial invasion [16]. The simultaneous detection of *C. trachomatis* and *U. parvum* was the most frequently observed. However, no specific pattern of co-detections is evident across the literature [18,29,67,68].

The *Protection+* health-promotion programme integrated the evaluation of the immunisation status for HAV. The main objective of this inclusion was to advise vaccination to all individuals at elevated risk of acquiring HAV infection through sexual intercourse who had not been vaccinated nor previously exposed to the virus. Although HAV infections are commonly transmitted through the ingestion of contaminated food and water, recent reports from developed countries have identified sexual transmission as a potential source of HAV infection, particularly in specific population groups, namely MSM [15,69]. A recent outbreak of HAV infection has been reported in Portugal, affecting mostly men aged between 18 and 44 years, identifying as MSM [70]. Nevertheless, according to the ECDC 2022 report, the mean rate of infection in Europe is low (one case per 100 000 population), and the notification rate in Portugal is even lower (0.3 per 100,000 population) [71]. Despite the fact that the HAV vaccine is available, in low-prevalence countries (such as Portugal), the National Vaccination Programme does not include this vaccine, which is usually reserved for individuals travelling to high-endemic regions.

In this study, HAV antibodies were detected in 26.1% of the participants. HAV-reported seroprevalence greatly varies depending on the studied population and geographic region. Seroprevalence values between 26.8% and 40.5% have been described in European countries [72,73], while values of 87.4% are reported for immigrants and refugees in Central Brazil [74]. According to the present results, a high proportion of the university community is not immune to HAV infection. It is therefore recommended that, despite the low endemicity of this infection in Portugal, particular attention should be given to high-risk groups.

The results of the present study reinforce the importance of screening for HIV, HBV, HCV and *T. pallidum* among university populations. These microorganisms are responsible for serious systemic infections, and screening programmes are crucial for an early detection and treatment initiation [60]. The benefits of *C. trachomatis*, *M. genitalium* and *N. gonorrhoea* screening are not evident in the available literature. Some authors suggest that screening programmes have little impact on the prevention of transmission and are associated with an increase in antimicrobial treatment and potential antimicrobial resistance [60]. Emerging antibiotic resistance has been reported for *N. gonorrhoeae* and *M. genitalium*, and a lower antibiotic sensitivity has been described for *C. trachomatis* [75,76]. Despite national as well as CDC guidelines not recommending the test-of-cure for these STIs [76], some of the participants diagnosed with STIs underwent a test-of-cure. The results obtained demonstrated the efficacy of the implemented treatment, indicating that the screening programme did not contribute to the emergence of antimicrobial resistance.

*C. trachomatis*, *M. genitalium* and *N. gonorrhoea* are frequently associated with self-limited mucosal infections that can sometimes be resolved by the immune system [60,77]. Nevertheless, serious health complications should not be dismissed. Pelvic inflammatory disease and infertility conditions occur as a complication of *C. trachomatis* infection, mainly in symptomatic patients [78]. Chlamydia screening policies vary across European countries, making difficult to clarify the role of screening programmes in preventing health complications [78]. The prevalence values reported in this study highlight the importance of implementing screening programmes in specific groups. Such a measure has also been suggested by other authors, based on studies conducted in STI high-risk populations [79]. The higher frequency of infections observed in younger age groups, reflects the importance of sexual education in preventing risky behaviours. In fact, the positive effect of STI awareness campaigns was evident in the present study, as the number of participants in the screening programme increased significantly after the two implemented campaigns.

## 5. Conclusions

The implementation of the health-promotion programme successfully achieved its main objective of raising awareness about STIs within the targeted university community. Despite the programme not accomplishing the desired level of engagement, probably due to the stigma still associated with STIs, the obtained results addressed the lack of knowledge regarding STIs within the Portuguese university population.

The present study reports a prevalence of STI higher than 10% in the studied population, with most patients being asymptomatic. *C. trachomatis* infection is the most frequent cause of STIs, followed by *M. genitalium* and *T. pallidum*. The implemented antimicrobial treatments were shown to be effective. Based on the obtained results, commensal microorganisms should be considered whenever STI symptoms are present in the absence of any known STI pathogenic agent.

The adherence to screening for STIs among students is low; however, the results of this programme emphasised the crucial importance of continuing to screening for the most common STIs and the benefits of the implementation of awareness campaigns on the prevention, transmission, symptoms and consequences of untreated STIs among the university community. The university learning environment is advantageous for introducing educational opportunities on critical aspects of sexual health, targeting students from different courses at key moments in their academic year, such as integration weeks and the weeks preceding academic festivities.

## Figures and Tables

**Table 1:** Demographic characteristics of participants in STI screening and obtained results. (HIV—Human Immunodeficiency Virus; HBV—Hepatitis B Virus; HCV—Hepatitis C Virus; HAV—Hepatitis A Virus).

Microorganism	Total		GENDER				AGE									
			Male		Female		15–19		20–24		25–34		35–44		≥45	
	Tested	Detected (%)	Tested	Detected (%)	Tested	Detected (%)	Tested	Detected (%)	Tested	Detected (%)	Tested	Detected (%)	Tested	Detected (%)	Tested	Detected (%)
*C. trachomatis*	475	40 (8.4%)	176	9 (5.1%)	299	31 (10.4%)	31	1 (3.2%)	243	26 (10.7%)	140	11 (7.9%)	44	2 (4.5%)	17	0 (0%)
*M. genitalium*	475	11 (2.3%)	176	6 (3.4%)	299	5 (1.7%)	31	0 (0%)	243	4 (1.6%)	140	6 (4.3%)	44	0 (0%)	17	1 (5.9%)
*T. pallidum*	345	7 (2.0%)	152	6 (3.9%)	193	1 (0.5%)	27	0 (0%)	184	4 (2.1%)	100	3 (2.9%)	27	0 (0%)	7	0 (0%)
*N. gonorrhoeae*	475	5 (1.1%)	176	2 (1.1%)	299	3 (1.0%)	31	1 (3.2%)	243	4 (1.6%)	140	0 (0%)	44	0 (0%)	17	0 (0%)
HIV	342	2 (0.6%)	150	2 (1.3%)	192	0 (0%)	27	0 (0%)	183	0 (0%)	97	1 (1.0%)	28	1 (3.6%)	7	0 (0%)
HBV	336	2 (0.6%)	143	2 (1.4%)	193	0 (0%)	27	0 (0%)	176	0 (0%)	98	2 (2.0%)	28	0 (0%)	7	0 (0%)
HCV	343	1 (0.3%)	150	0 (0%)	193	1 (0.5%)	27	0 (0%)	184	0 (0%)	98	0 (0%)	27	1 (3.7%)	7	0 (0%)
*T. vaginalis*	225	0 (0%)	60	0 (0%)	154	0 (0%)	6	0 (0%)	103	0 (0%)	74	0 (0%)	28	0 (0%)	14	0 (0%)
*U. parvum*	114	41 (36.0%)	41	5 (12.2%)	73	36 (49.3%)	3	1 (33.3%)	58	20 (34.5%)	37	15 (40.5%)	12	4 (33.3%)	4	1 (25.0%)
*U. urealyticum*	114	16 (14.0%)	41	3 (7.3%)	73	13 (17.9%)	3	1 (33.3%)	58	8 (13.8%)	37	6 (16.2%)	12	0 (0%)	4	1 (25.0%)
*M. hominis*	114	11 (9.6%)	41	0 (0%)	73	11 (15.1%)	3	0 (0%)	58	5 (8.6%)	37	5 (13.5%)	12	1 (8.3%)	4	0 (0%)
HAV *	295	*	130	*	165	*	26	*	165	*	80	*	20	*	4	*

* Evaluation of HAV infection only assessed previous immunisation and not the diagnosis of acute infection. These results are presented and discussed in a different section (Section 3.9).

**Table 2:** Cases of detection of multiple sexually transmitted agents. (HIV—Human Immunodeficiency Virus; HCV—Hepatitis C Virus).

Pathogenic Agent(s)	Commensal Agent(s)	Number of Cases
*C. trachomatis*; *M. genitalium*	-	1
*T. pallidum*; *M. genitalium*	-	1
*T. pallidum*; HIV	-	1
*C. trachomatis*	*U. parvum*	5
*C. trachomatis*	*U. urealyticum*; *M. hominis*	2
*C. trachomatis*	*U. parvum*; *U. urealyticum*; *M. hominis*	2
*C. trachomatis*	*U. parvum; M. hominis*	1
*C. trachomatis*	*U. urealyticum*	1
*M. genitalium*	*U. parvum*; *U. urealyticum*; *M. hominis*	1
HCV	*U. parvum*	1
	*U. parvum; M. hominis*	3
	*U. parvum*; *U. urealyticum*; *M. hominis*	2
	*U. parvum*; *U. urealyticum*	1

## Data Availability

The original contributions presented in the study are included in the article, further inquiries can be directed to the corresponding author.

## References

[1] Rowley J., Vander Hoorn S., Korenromp E., Low N., Unemo M., Abu-Raddad L.J., Chico R.M., Smolak A., Newman L., Gottlieb S. (2019). Chlamydia, Gonorrhoea, Trichomoniasis and Syphilis: Global Prevalence and Incidence Estimates, 2016. Bull. World Health Organ..

[2] McHaro R.D., Kisinda A., Njovu L., McHaro M., Mbwilo F., Mihale G., Komba B., Andrew E., Mayaud P., Kroidl A. (2022). Prevalence of and Risk Factors Associated with HIV, Herpes Simplex Virus-Type 2, *Chlamydia trachomatis* and *Neisseria gonorrhoeae* Infections Among 18–24 Year Old Students Attending Higher Learning Institutions in Mbeya-Tanzania. PLoS ONE.

[3] Cannovo N., Bianchini E., Gironacci L., Garbati E., Di Prospero F., Cingolani M., Scendoni R., Fedeli P. (2024). Sexually Transmitted Infections in Adolescents and Young Adults: A Cross Section of Public Health. Int. J. Environ. Res. Public Health.

[4] WHO Sexually Transmitted Infections (STIs). World Health Organization: WHO. https://www.who.int/News-Room/Fact-Sheets/Detail/Sexually-Transmitted-Infections-(Stis).

[5] Tuddenham S., Hamill M.M., Ghanem K.G. (2022). Diagnosis and Treatment of Sexually Transmitted Infections. JAMA.

[6] Chapagain N., Chapagain S. (2023). Need for Hepatitis B Vaccination in Medical Students. J. Nepal Med. Assoc..

[7] Rahangdale L., Mungo C., O’Connor S., Chibwesha C.J., Brewer N.T. (2022). Human Papillomavirus Vaccination and Cervical Cancer Risk. BMJ.

[8] Cole S. (2020). Herpes Simplex Virus. Nurs. Clin. N. Am..

[9] Barbier F., Mer M., Szychowiak P., Miller R.F., Mariotte É., Galicier L., Bouadma L., Tattevin P., Azoulay É. (2020). Management of HIV-Infected Patients in the Intensive Care Unit. Intensive Care Med..

[10] Sienkiewicz L., Thomas Y., Reynoso A., Munson E. (2023). Incidence and Laboratory Diagnosis of Sexually-Transmitted Infections among University Students in a High-Prevalence Community. J. Am. Coll. Health.

[11] Zhang Z., Zong X., Bai H., Fan L., Li T., Liu Z. (2023). Prevalence of *Mycoplasma genitalium* and *Chlamydia trachomatis* in Chinese Female with Lower Reproductive Tract Infection: A Multicenter Epidemiological Survey. BMC Infect. Dis..

[12] Karagoz A., Tombuloglu H., Alsaeed M., Tombuloglu G., AlRubaish A.A., Mahmoud A., Smajlović S., Ćordić S., Rabaan A.A., Alsuhaimi E. (2023). Monkeypox (Mpox) Virus: Classification, Origin, Transmission, Genome Organization, Antiviral Drugs, and Molecular Diagnosis. J. Infect. Public Health.

[13] Al-Tammemi A.B., Sallam M., Rebhi A., Soliman L., Al Sarayrih L., Tarhini Z., Abutaima R., Aljaberi M.A., Barakat M. (2022). The Outbreak of Ebola Virus Disease in 2022: A Spotlight on a Re-Emerging Global Health Menace. Narra J..

[14] Major C.G., Paz-Bailey G., Hills S.L., Rodriguez D.M., Biggerstaff B.J., Johansson M. (2021). Risk Estimation of Sexual Transmission of Zika Virus—United States, 2016–2017. J. Infect. Dis..

[15] Migueres M., Lhomme S., Izopet J. (2021). Hepatitis A: Epidemiology, High-Risk Groups, Prevention and Research on Antiviral Treatment. Viruses.

[16] Van Gerwen O.T., Muzny C.A., Marrazzo J.M. (2022). Sexually Transmitted Infections and Female Reproductive Health. Nat. Microbiol..

[17] Foschi C., Zagarrigo M., Belletti M., Marangoni A., Re M.C., Gaspari V. (2020). Genital and Extra-Genital *Chlamydia trachomatis* and *Neisseria gonorrhoeae* Infections in Young Women Attending a Sexually Transmitted Infections (STI) Clinic. New Microbiol..

[18] Grad A.I., Vică M.L., Ungureanu L., Siserman C.V., Tătaru A.D., Matei H.V. (2020). Assessment of STI Screening in Romania Using a Multiplex PCR Technique. J. Infect. Dev. Ctries..

[19] Spindola T., de Melo L.D., Brandão J.d.L., de Oliveira D.C., Marques S.C., Arreguy-Sena C., Pinto P.F. (2023). Social Representation of Young People in Higher Education about Sexually Transmitted Infections. Rev. Bras. Enferm..

[20] Rodrigues R., Sousa C., Vale N. (2022). *Chlamydia trachomatis* as a Current Health Problem: Challenges and Opportunities. Diagnostics.

[21] Scaglione E., Mantova G., Caturano V., Fanasca L., Carraturo F., Farina F., Pagliarulo C., Vitiello M., Pagliuca C., Salvatore P. (2022). Molecular Epidemiology of Genital Infections in Campania Region: A Retrospective Study. Diagnostics.

[22] ECDC European Centre for Disease Prevention and Control (2021). Technical Report: Technologies, Strategies and Approaches for Testing Populations at Risk of Sexually Transmitted Infections in the EU/EEA.

[23] Ezeanya-Bakpa C.C., Agbakoba N.R., Oguejiofor C.B., Enweani-Nwokelo I.B. (2021). Sequence Analysis Reveals Asymptomatic Infection with *Mycoplasma hominis* and *Ureaplasma urealyticum* Possibly Leads to Infertility in Females: A Cross-Sectional Study. Int. J. Reprod. Biomed..

[24] Liu W., Yang T., Kong Y., Xie X., Ruan Z. (2024). *Ureaplasma* Infections: Update on Epidemiology, Antimicrobial Resistance, and Pathogenesis. Crit. Rev. Microbiol..

[25] Stol K., Jans J., De Bruin L.O., Unger W., Van Rossum A. (2021). Perinatal Infections with *Ureaplasma*. Pediatr. Infect. Dis. J..

[26] DGS Direção Geral de Saúde (2014). Norma 058/2011; Diagnóstico e Rastreio Laboratorial Da Infeção Pelo Vírus Da Imunodeficiência Humana (VIH).

[27] DGS Direção Geral de Saúde (2017). Norma 027/2017; Avaliação Diagnóstica Da Infeção Por Vírus Da Hepatite C.

[28] DGS Direção Geral de Saúde (2017). Norma 003/2017; Hepatite A.

[29] Cutoiu A., Boda D. (2023). Prevalence of *Ureaplasma urealyticum*, *Mycoplasma hominis* and *Chlamydia trachomatis* in Symptomatic and Asymptomatic Patients. Biomed. Rep..

[30] Leli C., Mencacci A., Latino M.A., Clerici P., Rassu M., Perito S., Castronari R., Pistoni E., Luciano E., De Maria D. (2018). Prevalence of Cervical Colonization by *Ureaplasma parvum*, *Ureaplasma urealyticum*, *Mycoplasma hominis* and *Mycoplasma genitalium* in Childbearing Age Women by a Commercially Available Multiplex Real-Time PCR: An Italian Observational Multicentre Study. J. Microbiol. Immunol. Infect..

[31] Tadera K., Kitagawa H., Kitano H., Hara T., Kashiyama S., Nomura T., Omori K., Shigemoto N., Yokozaki M., Ohge H. (2023). Prevalence of *Mycoplasma hominis*, *Ureaplasma urealyticum*, and *Ureaplasma parvum* Detection in Urine and Respiratory Tract Samples in Hiroshima, Japan. Heliyon.

[32] Karim S., Bouchikhi C., Bouchikhi C., Banani A., Fatemi H.E.L., Souho T., Erraghay S., Bennani B., Bennani B. (2020). Detection of *Ureaplasma* Biovars and Subtyping of *Ureaplasma parvum* among Women Referring to a University Hospital in Morocco. Infect Dis Obstet Gynecol.

[33] Wang Q.Y., Li R.H., Zheng L.Q., Shang X.H. (2016). Prevalence and Antimicrobial Susceptibility of *Ureaplasma urealyticum* and *Mycoplasma hominis* in Female Outpatients, 2009–2013. J. Microbiol. Immunol. Infect..

[34] Molla G., Desalegn A., Tigu F. (2021). Prevalence of Gonorrhea and Associated Knowledge, Attitude and Risky Behaviors and Preventive Practices Among High School Students: A Cross-Sectional Study. J. Community Health.

[35] Bashmaq S.M., Ahmadi A., Mohsenpour B., Rahmani K., Arasteh M., Alizadeh N.S., Babahajian A., Advay S., Abbaszadeh A. (2024). Prevalence of HIV, HBV, HCV, HPV and Syphilis among Female Sex Workers in Kurdistan, West of Iran. Casp. J. Intern. Med..

[36] Nijhuis R.H.T., Duinsbergen R.G., Pol A., Godschalk P.C.R. (2021). Prevalence of *Chlamydia trachomatis*, *Neisseria gonorrhoeae*, *Mycoplasma genitalium* and *Trichomonas vaginalis* Including Relevant Resistance-Associated Mutations in a Single Center in the Netherlands. Eur. J. Clin. Microbiol. Infect. Dis..

[37] ECDC European Centre for Disease Prevention and Control (2024). Chlamydia. Annual Epidemiological Report for 2022.

[38] Klavs I., Milavec M., Berlot L., Kustec T., Grgič-Vitek M., Lavtar D., Zaletel M., Golle A., Duh D., Čretnik T.Ž. (2022). Prevalence of Sexually Transmitted Infections with *Chlamydia trachomatis*, *Neisseria gonorrhoeae*, *Mycoplasma genitalium* and *Trichomonas vaginalis*: Findings from the National Survey of Sexual Lifestyles, Attitudes and Health, Slovenia, 2016 to 2017. Eurosurveillance.

[39] Hedley P.L., Hoffmann S., Lausten-Thomsen U., Voldstedlund M., Bjerre K.D., Hviid A., Krebs L., Jensen J.S., Christiansen M. (2022). A Nationwide Observational Study of *Chlamydia trachomatis* Infections in Denmark during the COVID-19 Pandemic. Acta Derm. Venereol..

[40] Desdorf R., Andersen N.M., Chen M. (2021). *Mycoplasma genitalium* Prevalence and Macrolide Resistance-Associated Mutations and Coinfection with *Chlamydia trachomatis* in Southern Jutland, Denmark. APMIS.

[41] Ribeiro A.A., Saddi V.A., Carneiro M.A., Figueiredo-Alves R.R., da Silva Barros N.K., de Almeida Carvalho K.P., do Nascimento Tavares S.B., de Araújo Teles S., D’Alessandro W.B., Rabelo-Santos S.H. (2020). Human Papillomavirus and *Chlamydia trachomatis* Infections in Adolescents and Young Women: Prevalence and Risk Factors. Diagn. Cytopathol..

[42] DGS Direção Geral de Saúde (2021). Diário Da República, 2.^a^ Série: Despacho N^o^ 1150-2021.

[43] Gnanadurai R., Fifer H. (2020). *Mycoplasma genitalium*: A Review. Microbiology.

[44] Uysal H., Koksal M.O., Sarsar K., Ilktac M., Isik Z., Akgun Karapinar D.B., Demirci M., Ongen B., Buyukoren A., Kadioglu A. (2023). Prevalence of *Chlamydia trachomatis*, *Neisseria gonorrhoeae*, and *Mycoplasma genitalium* among Patients with Urogenital Symptoms in Istanbul. Healthcare.

[45] Yusuf E., Mertens K., Van Lisdonk N., Houwen C., Thai K.T.D. (2023). Epidemiology of *Mycoplasma genitalium* and *Trichomonas vaginalis* in the Primary Health Care Setting in the Netherlands. Epidemiol. Infect..

[46] Perry M.D., Jones S., Bertram A., de Salazar A., Barrientos-Durán A., Schiettekatte G., Lewinski M., Arcenas R., Hansra A., Njoya M. (2023). The Prevalence of *Mycoplasma genitalium* (MG) and *Trichomonas vaginalis* (TV) at Testing Centers in Belgium, Germany, Spain, and the UK Using the Cobas TV/MG Molecular Assay. Eur. J. Clin. Microbiol. Infect. Dis..

[47] Minetti C., Rocha M., Duque L.M., Meireles P., Correia C., Cordeiro D., João I., Manita C., Soeiro S., Santos J.A. (2024). Orogenital and Anal Infection by *Chlamydia trachomatis*, *Neisseria gonorrhoeae*, *Mycoplasma genitalium*, and Other Sexually Transmitted Infections in Men Who Have Sex with Men in Lisbon. Int. J. STD AIDS.

[48] Silva J., Cerqueira F., Teixeira A.L., Bicho M.C., Campainha R., Amorim J., Medeiros R. (2018). Genital Mycoplasmas and Ureaplasmas in Cervicovaginal Self-Collected Samples of Reproductive-Age Women: Prevalence and Risk Factors. Int. J. STD AIDS.

[49] Spiller O.B., Rees C.L., Morris D.J., Davies R.L., Jones L.C. (2020). *Mycoplasma genitalium* Prevalence in Welsh Sexual Health Patients: Low Antimicrobial Resistance Markers and No Association of Symptoms to Bacterial Load. Microb. Pathog..

[50] ECDC European Centre for Disease Prevention and Control (2024). Syphilis. Annual Epidemiological Report for 2022.

[51] ECDC European Centre for Disease Prevention and Control (2024). Gonorrhoea. Annual Epidemiological Report for 2022.

[52] Herrero M., Broner S., Cruells A., Esteve S., Ferré L., Mendioroz J., Jané M., Ciruela P., Benítez M.Á., Bosch J. (2023). Epidemiology and Antimicrobial Resistance Profile of *Neisseria gonorrhoeae* in Catalonia, Spain, 2016–2019. Eur. J. Clin. Microbiol. Infect. Dis..

[53] ECDC European Centre for Disease Prevention and Control, WHO Office for Europe (2023). HIV/AIDS Surveillance in Europe—2022 Data.

[54] Razavi-Shearer D., Gamkrelidze I., Pan C., Jia J., Berg T., Gray R., Lim Y.-S., Chen C.-J., Ocama P., Desalegn H. (2023). Global Prevalence, Cascade of Care, and Prophylaxis Coverage of Hepatitis B in 2022: A Modelling Study. Lancet Gastroenterol. Hepatol..

[55] Cui F., Blach S., Manzengo Mingiedi C., Gonzalez M.A., Sabry Alaama A., Mozalevskis A., Séguy N., Rewari B.B., Chan P.-L., Le L. (2023). Global Reporting of Progress towards Elimination of Hepatitis B and Hepatitis C. Lancet Gastroenterol. Hepatol..

[56] Nguyen M.H., Wong G., Gane E., Kao J.-H., Dusheiko G. (2020). Hepatitis B Virus: Advances in Prevention, Diagnosis, and Therapy. Clin. Microbiol. Rev..

[57] ECDC European Centre for Disease Prevention and Control (2024). Hepatitis B. Annual Epidemiological Report for 2022.

[58] Lakoh S., García-Tardón N., Adekanmbi O., van der Valk M., Smith S.J., Grobusch M.P. (2021). Prevalence of Viral Hepatitis B and C in Sierra Leone—Current Knowledge and Knowledge Gaps: A Narrative Review. Trans. R. Soc. Trop. Med. Hyg..

[59] ECDC European Centre for Disease Prevention and Control (2024). Hepatitis C. Annual Epidemiological Report for 2022.

[60] Kenyon C., Herrmann B., Hughes G., de Vries H.J.C. (2023). Management of Asymptomatic Sexually Transmitted Infections in Europe: Towards a Differentiated, Evidence-Based Approach. Lancet Reg. Health Eur..

[61] Silva J., Cerqueira F., Teixeira A.L., Campainha R., Amorim J., Medeiros R. (2021). Prevalence of *Neisseria gonorrhoeae* and *Trichomonas vaginalis* in Portuguese Women of Childbearing Age. J. Obstet. Gynaecol..

[62] Van Gerwen O.T., Camino A.F., Sharma J., Kissinger P.J., Muzny C.A. (2021). Epidemiology, Natural History, Diagnosis, and Treatment of *Trichomonas vaginalis* in Men. Clin. Infect. Dis..

[63] Kebbi-Beghdadi C., Aeby S., Baud D., Greub G. (2022). Evaluation of a Multiplex Real-Time PCR Assay for Detecting *Chlamydia trachomatis* in Vaginal Samples. Diagnostics.

[64] Kasprzykowska U., Sobieszczańska B., Duda-Madej A., Secewicz A., Nowicka J., Gościniak G. (2018). A Twelve–Year Retrospective Analysis of Prevalence and Antimicrobial Susceptibility Patterns of *Ureaplasma* spp. and *Mycoplasma hominis* in the Province of Lower Silesia in Poland. Eur. J. Obstet. Gynecol. Reprod. Biol..

[65] Lee J.Y., Yang J.S. (2020). Prevalence and Antimicrobial Susceptibility of *Mycoplasma hominis* and *Ureaplasma* Species in Nonpregnant Female Patients in South Korea Indicate an Increasing Trend of Pristinamycin-Resistant Isolates. Antimicrob. Agents Chemother..

[66] Carneiro F.P., Darós A.C., Darós A.C.M., De Castro T.M.M.L., De Vasconcelos Carneiro M., Fidelis C.R., Vilioni M.V., Da Costa Matsunaga M.E., Sidou J.M.O., Chaves M.A.L.D. (2020). Cervical Cytology of Samples with *Ureaplasma urealyticum*, *Ureaplasma parvum*, *Chlamydia trachomatis*, *Trichomonas vaginalis*, *Mycoplasma hominis*, and *Neisseria gonorrhoeae* Detected by Multiplex PCR. BioMed Res. Int..

[67] Cai S., Pan J., Duan D., Yu C., Yang Z., Zou J. (2020). Prevalence of *Ureaplasma urealyticum*, *Chlamydia trachomatis*, and *Neisseria gonorrhoeae* in Gynecological Outpatients, Taizhou, China. J. Clin. Lab. Anal..

[68] Esen B., Gozalan A., Sevindi D.F., Demirbas A., Onde U., Erkayıran U., Karakoc A.E., Hasçiçek A.M., Ergün Y., Adiloglu A.K. (2017). *Ureaplasma urealyticum*: Presence among Sexually Transmitted Diseases. Jpn. J. Infect. Dis..

[69] Abutaleb A., Kottilil S. (2020). Hepatitis A: Epidemiology, Natural History, Unusual Clinical Manifestations, and Prevention. Gastroenterol. Clin. N. Am..

[70] Rosendal E., von Schreeb S., Gomes A., Lino S., Grau-Pujol B., Magalhães S., Ricoca Peixoto V., Roque C., Moreno J., Maltez F. (2024). Ongoing Outbreak of Hepatitis A Associated with Sexual Transmission among Men Who Have Sex with Men, Portugal, October 2023 to April 2024. Eurosurveillance.

[71] ECDC European Centre for Disease Prevention and Control (2024). Hepatitis A. Annual Epidemiological Report for 2022.

[72] Vilibic-Cavlek T., Zidovec-Lepej S., Ferenc T., Savic V., Nemeth-Blazic T., Vujica Ferenc M., Bogdanic M., Vilibic M., Simunov B., Janev-Holcer N. (2023). Seroprevalence Trends and Molecular Epidemiology of Viral Hepatitis in Croatia. Life.

[73] Mossong J., Putz L., Patiny S., Schneider F. (2006). Seroepidemiology of Hepatitis A and Hepatitis B Virus in Luxembourg. Epidemiol. Infect..

[74] da Costa e Silva G.R., Martins T.L.S., de Almeida Silva C., Caetano K.A.A., dos Santos Carneiro M.A., e Silva B.V.D., Pacheco L.R., Villar L.M., de Paula V.S., Martins R.M.B. (2022). Hepatitis A and E among Immigrants and Refugees in Central Brazil. Rev. Saude Publica.

[75] Wind C.M., Schim van der Loeff M.F., Unemo M., Schuurman R., van Dam A.P., de Vries H.J.C. (2016). Time to Clearance of *Chlamydia trachomatis* RNA and DNA after Treatment in Patients Coinfected with *Neisseria gonorrhoeae*—A Prospective Cohort Study. BMC Infect. Dis..

[76] Dalby J., Stoner B. (2022). Sexually Transmitted Infections: Updates From the 2021 CDC Guidelines. Am. Fam. Phys..

[77] Geisler W.M., Morrison S.G., Doemland M.L., Iqbal S.M., Su J., Mancevski A., Hook E.W., Morrison R.P. (2012). Immunoglobulin-Specific Responses to Chlamydia Elementary Bodies in Individuals with and at Risk for Genital Chlamydial Infection. J. Infect. Dis..

[78] Alexiou Z.W., Hoenderboom B.M., Hoebe C.J.P.A., Dukers-Muijrers N.H.T.M., Götz H.M., van der Sande M.A.B., de Vries H.J.C., den Hartog J.E., Morré S.A., van Benthem B.H.B. (2024). Reproductive Tract Complication Risks Following *Chlamydia trachomatis* Infections: A Long-Term Prospective Cohort Study from 2008 to 2022. Lancet Reg. Health Eur..

[79] Rondeau P., Valin N., Decré D., Girard P.M., Lacombe K., Surgers L. (2019). *Chlamydia trachomatis* Screening in Urine among Asymptomatic Men Attending an STI Clinic in Paris: A Cross-Sectional Study. BMC Infect. Dis..

